# Drivers of metacommunity structure diverge for common and rare Amazonian tree species

**DOI:** 10.1371/journal.pone.0188300

**Published:** 2017-11-20

**Authors:** Polyanna da Conceição Bispo, Heiko Balzter, Yadvinder Malhi, J. W. Ferry Slik, João Roberto dos Santos, Camilo Daleles Rennó, Fernando D. Espírito-Santo, Luiz E. O. C. Aragão, Arimatéa C. Ximenes, Pitágoras da Conceição Bispo

**Affiliations:** 1 Leicester Institute for Space and Earth Observation, Centre for Landscape and Climate Research, Department of Geography, University of Leicester, Leicester, United Kingdom; 2 NERC, National Centre for Earth Observation at the University of Leicester, Leicester, United Kingdom; 3 Environmental Change Institute, School of Geography and the Environment, University of Oxford, Oxford, United Kingdom; 4 Faculty of Science, Universiti Brunei Darussalam, Gadong, Brunei; 5 Remote Sensing Division, National Institute for Space Research (INPE), São José dos Campos, Brazil; 6 Image Processing Division, National Institute for Space Research (INPE), São José dos Campos, Brazil; 7 Lancaster Environment Centre, Lancaster University, Lancaster, United Kingdom; 8 College of Life and Environmental Sciences, University of Exeter, Exeter, United Kingdom; 9 Laboratory of Systems Ecology and Resource Management, Department of Biology of Organisms, Université Libre de Bruxelles, Brussels, Belgium; 10 Laboratory of Plant Biology and Nature Management, Ecology & Biodiversity, Vrije Universiteit Brussel, Brussels, Belgium; 11 Department of Biological Sciences, Faculty of Sciences and Languages of Assis, State University of São Paulo (UNESP), Assis, Brazil; Chinese Academy of Forestry, CHINA

## Abstract

We analysed the flora of 46 forest inventory plots (25 m x 100 m) in old growth forests from the Amazonian region to identify the role of environmental (topographic) and spatial variables (obtained using PCNM, Principal Coordinates of Neighbourhood Matrix analysis) for common and rare species. For the analyses, we used multiple partial regression to partition the specific effects of the topographic and spatial variables on the univariate data (standardised richness, total abundance and total biomass) and partial RDA (Redundancy Analysis) to partition these effects on composition (multivariate data) based on incidence, abundance and biomass. The different attributes (richness, abundance, biomass and composition based on incidence, abundance and biomass) used to study this metacommunity responded differently to environmental and spatial processes. Considering standardised richness, total abundance (univariate) and composition based on biomass, the results for common species differed from those obtained for all species. On the other hand, for total biomass (univariate) and for compositions based on incidence and abundance, there was a correspondence between the data obtained for the total community and for common species. Our data also show that in general, environmental and/or spatial components are important to explain the variability in tree communities for total and common species. However, with the exception of the total abundance, the environmental and spatial variables measured were insufficient to explain the attributes of the communities of rare species. These results indicate that predicting the attributes of rare tree species communities based on environmental and spatial variables is a substantial challenge. As the spatial component was relevant for several community attributes, our results demonstrate the importance of using a metacommunities approach when attempting to understand the main ecological processes underlying the diversity of tropical forest communities.

## Introduction

Throughout the history of studying ecology, researchers have sought to understand the effects of environmental and spatial processes on biodiversity. This quest has led to the collection of a large number of datasets and the formulation of competing theories, such as niche and neutral theories. The niche theory was developed during the 20^th^ century and has been one of the most important theoretical approaches in ecology. This theory assumes that the species in a community are different and that the combination of available resources and environmental conditions determines the local diversity [1]. The coexistence of species within a community, therefore, can be mainly explained by the way in which species with different resource needs and environmental requirements partition existing niches. In this context, it is expected that communities structured by niche-related processes have similar values for local attributes (e.g., species richness, species abundance, biomass and composition) in similar habitat patches. In contrast with the niche theory, the neutral theory is based on the functional equivalence among species and considers dispersion and demographic stochasticity as central phenomena [2,3]. The neutral theory considers that the diversity of a community is a result of the dynamic balance between immigration and extinction [4,5]. According to this theory, it is expected that similar values for local attributes of a community in a particular patch can be determined by the influence of communities of nearby patches, stressing the importance of spatial processes.

In the past few decades, the neutral theory has brought new force to discussions about the processes that determine diversity in communities. In this debate, the proponents of the niche theory have reacted strongly against the assumption that species are equivalent, which is advocated by the neutral theory. For their part, supporters of the neutral theory argue that, in fact, they do not believe that species are equivalent but that this assumption (in addition to the use of stochastic elements) allows the building of simple models with good predictive ability [6]. The supporters of the two theories are usually on opposite sides [5–9]; however, there are researchers who have tried to reconcile them [10]. Although the neutral theory is controversial because it radicalises the assumptions [5], it brings important elements such as the limitation of dispersion and ecological drift, which, along with the niche theory, can help us understand the processes involved in the diversity of species in communities. Perhaps the best way to consider these two theories is as representative of two extremes along a *continuum* [10], among which communities are structured by the relative balance between the mechanisms emphasised by the niche (e.g. environmental filters) and neutral (e.g. dispersion and ecological drift) theories.

Megadiverse tropical forests can be important model ecosystems for understanding the relative roles of environmental and spatial variables on communities. Efforts to understand these roles may help clarify the importance of different processes in maintaining the species diversity of these forests. In terms of the niche theory, variables representative of topography are likely to play an important role in local environmental conditions, which may determine the species diversity, functional and structural attributes and composition of tree communities [11–14]. One advantage of using topography in vegetation studies is that it can be assessed at large scales by remote sensing. Moreover, topography is considered a good surrogate for several important variables of vegetation structure that would be difficult to measure on a larger scale, including nutrient availability, soil moisture and texture, and insolation [13].

The spatial processes, despite their known importance, were long ignored in ecological studies, and until the 1990s, the main focus of ecology was the study of niche-related processes [15]. Since then, spatial processes have been strongly embedded in ecology [16] and new methods and analytical strategies have been proposed, generating a new body of knowledge about the structuring factors of communities [17–19]. This knowledge, along with other information (functional, phylogenetic, etc.), has provided a much more detailed understanding of the processes involved in community structure [20–22], supporting the search for better conservation and biodiversity monitoring strategies [23]. Understanding the role of spatial component in the community structure at different landscape scales is essential, especially given the current scenario of rapid biodiversity loss due to habitat degradation and fragmentation [24].

Metacommunity can be defined by groups of communities that are connected to each other by the movements of individuals of different species [25,26]. This approach recognises that communities are not isolated entities. The metacommunity theory has benefited from discussions regarding niche and neutral theories, and both have helped to understand the effects of environmental and spatial variables on the diversity of species in communities [25,26]. According to the theory, the group of species occurring in a community is determined both by a combination of local factors (interactions among species and interactions of species with local environmental factors) and by the ability of the species to reach that community (by dispersion) [25,27]. In metacommunities structured by the principles of niche theory, it is expected that the environmental component plays a more important role. In contrast, in metacommunities subject to the principles of neutral theory, it is expected that the spatial component plays a more important role. The metacommunity theory encompasses four main models (species sorting, mass effect, patch dynamics and neutral models), which represent points along a *continuum* formed by different combinations of environmental and dispersal processes in different ecological scenarios [25,26,28,29].

A remarkable feature of most communities is the presence of a few common species and many rare species [30,31]. Rare and common species can respond differently to ecological processes [32], depending on the features of the organisms (e.g., competition and dispersion capacities) and the spatial temporal dynamics. For example, based on the niche theory, Tokeshi [15] proposed the composite niche model, arguing that more than one process may be acting on the community. According to this model, common species should fit any model of niche apportionment, while rare species should fit a random assortment model. On the other hand, Siqueira et al. [31] studied metacommunities of aquatic macroinvertebrates and showed that common and rare species responded similarly and that both were mainly structured by niche processes.

The analysis of common and rare species allows the testing of some hypotheses about the processes involved in the structuring of metacommunities [31,33]. Empirical studies have shown that most metacommunities are structured principally by niche processes [28,31]. Our first hypothesis is that niche processes are more important for structuring the metacommunity studied [31], at least for the common species, which we expect to be most affected by competition [34]. In the case of rare species, we expect spatial variables to be more relevant, as these species can be more affected by ecological drift [34]. On the other hand, taking into account that habitat generalist and habitat specialist species differ in terms of population dynamics, we also propose an alternative hypothesis [31,35]. While generalist species occupy habitats with broad environmental variation, specialist species preferentially occupy habitats with specific environmental characteristics, which are generally rare in the landscape [35,36]. In this context, assuming that common species are habitat generalists and rare species are habitat specialists, our alternative hypothesis is that spatial component is more important for common species, while environmental component is crucial for rare species [35].

To test these hypotheses and to identify the role of environmental (topographic) and spatial variables for common and rare species, we analysed the flora of 46 forest inventory plots in the old growth forests of the eastern Amazon region. These analyses were based on vegetation data collected in the field and topographic variables obtained by remote sensing data.

## Material and methods

### Study area

**Fig 1:**
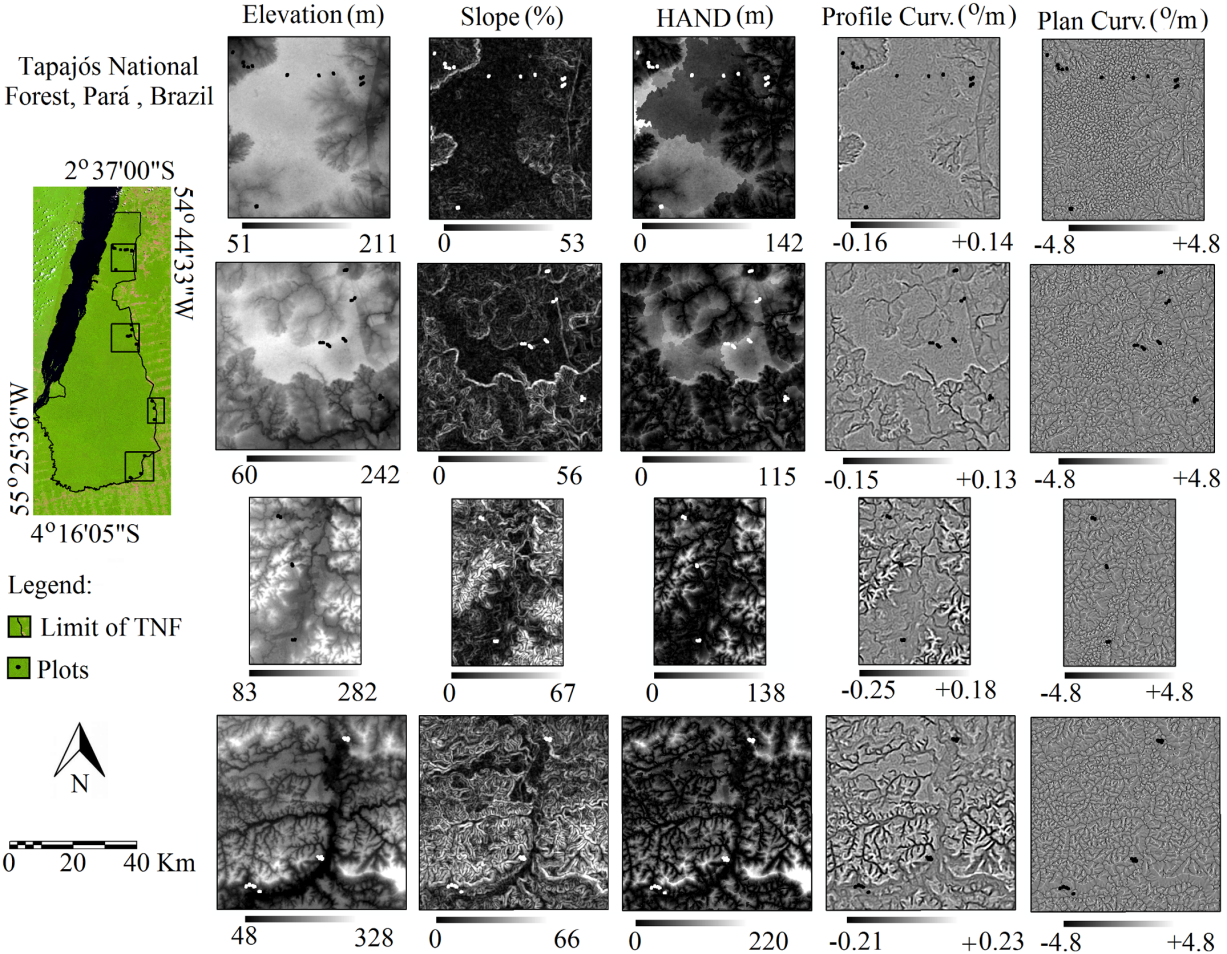
Study area in the Tapajós National Forest (TNF), Pará State, Brazil, with details of the five geomorphometric variables (elevation, slope, HAND, profile curvature and plan curvature) of the four areas where the 46 plots were distributed.

This study was conducted in the Tapajós National Forest (TNF). The TNF is a large protected area of approximately 545,000 ha, located in Amazon biome, western part of Pará State, Brazil (Fig 1). This area has an average annual temperature of 25.5°C and average annual rainfall of 1,820 mm. The local topography ranges from flat to strongly undulating terrain. Predominant soil types in the area are dystrophic oxisol (US classification) or dystrophic yellow latosol (Brazil classification) and red-yellow podzol. Vegetation is mainly ombrophilous dense forest and ombrophilous open forest [37].

We sampled 46 forest inventory plots (Fig 1) of 25 x 100 m in the TNF. Our plots represented a sampling area of 11.5 ha. We installed the plots in different vegetal typologies and topographies [12] using the phyto-ecologic classes from the RADAM-BRASIL project [38]. Our plots encompassed different floristic and geomorphological characteristics [38]. We sampled and identified all individual trees with diameter at breast height (DBH) ≥ 10 cm. The abundance can be represented by the number of individuals and by biomass. To differentiate these two attributes throughout the text, hereafter, we use “abundance” to refer to the number of individuals and “biomass” to refer to above ground biomass. The biomass was calculated by the allometric equation [39], using the measurements of DBH and TH (total height).

$$\text{Biomass}=0.044*{\left(({\text{DBH}}^{2})*\text{TH}\right)}^{0.9719}$$

### Topographic data derived from SRTM

We used geomorphometric attributes (elevation, slope, profile curvature and plane curvature) from the Brazilian Geomorphometrics Database (TOPODATA) [40]. TOPODATA is based on the SRTM (Shuttle Radar Topography Mission-version 1, NASA, 2006) and has different neighbourhood operations to calculate geomorphometric variables [40]. TOPODATA is free, and the layers are easily accessible (http://www.dsr.inpe.br/topodata/acesso.php). We also used the vertical distance to the nearest drainage or HAND (height above the nearest drainage). HAND was derived from the SRTM and describes the vertical distance of each point regarding the nearest drainage channel detected by remote sensing [41]. All data used in this study have a spatial resolution of 30 m (Table 1).

**Table 1:** Definitions of the topographic variables used in this study.

Topographic variables	Description
Elevation (*h)*	Terrain altitude. This is related to the altitude distribution of soil and climate, determining different landscape vegetation patterns.
Slope (*G)*	Inclination angle of the local surface. This has a direct effect on the balance between soil water infiltration and surface runoff and controls the intensity of flows of matter and insolation. This set of factors results in environments with different physical and biological characteristics, allowing the establishment of different types of vegetation.
Profile curvature (*kv*)	Concave/convex character of the terrain. This characterizes the land surface, which is directly associated with hydrological and transport properties and may directly influence the distribution and development of vegetation.
Plan curvature (*kh)*	Divergent/convergent character of flows of matter on the ground when analysed on a horizontal projection. As with the profile curvature, the plan curvature characterises the land surface, which is directly associated with hydrological and transport properties and may indirectly influence vegetation.
Height above the nearest drainage (*HAND*)	Describes the vertical distance of each point regarding the nearest drainage channel. It can reveal the local water table conditions (the lower the HAND value, the closer the water table is of the surface).

Topographic variables obtained on the basis of the SRTM have been used to explain or predict the properties of vegetation [42,43]. These studies have helped in the understanding of the effects of topography on the distribution of different types of vegetation [44–48], floristic composition [12,49] and forest structure [14,50], particularly in tropical areas.

### Data analysis

We defined common and rare species using the criterion of the inflection point of the curve of species abundance (or species biomass) [31]. We defined the inflection point visually; species left of this point were considered as common and species to the right as rare [31]. As matrices with different amounts of information can affect the results, we made comparisons considering the same information content. First, the information content of the matrices of common and rare species was calculated based on the binomial variance of the incidence matrix, *∑p*_*i*_*(1-p*_*i*_*)*, where *p*_*i*_ is the proportion of plots occupied by i^th^ species [31,51]. As the matrix of rare species had a higher information content, we removed rare species, following species rank, until this matrix had the same information content as that of common species.

After defining common and rare species with the same information content, we performed data analyses considering univariate and multivariate community attributes. The univariate attributes were standardised richness (residuals of regression between abundance and richness), total abundance (sum of the abundance of the species per plot) and total biomass (total biomass of the species per plot). The multivariate attributes (species x plots) were represented by three different matrices of composition (1. composition based on incidence or presence-absence; 2. composition based on abundance; and 3. composition based on biomass).

The richness of species is usually positively correlated with abundance. As abundance can explain part of the variation in richness, without due caution, we can erroneously conclude that similar factors are important in explaining both community attributes. Disentangling richness from abundance is necessary to understand the real effect of topography on richness (free of abundance). For this reason, we used the residuals of the regression between abundance and richness as a standardised measure of richness (standardised richness). In this case, the residuals indicate the part of the variation in richness that cannot be explained by abundance, in other words, richness free of abundance.

In general, the community matrix (species x plots) based on abundance has many zeros, which is a problem for multivariate analysis based on Euclidian distances, such as Principal Component Analysis (PCA) and Redundancy Analysis (RDA). A strategy to minimise this problem is using the Hellinger transformation [52]. In the case of composition based on abundance, prior to the analyses, we transformed the data matrix using the Hellinger method [53].

For the analyses, we used a multiple partial regression to partition the specific effects of the topographic and spatial variables on the univariate response variables (standardised richness, abundance and total biomass), and we used partial RDA to partition these effects on the multivariate response matrices represented by incidence, abundance and biomass [17,54,55]. The RDA is a direct gradient analysis based on multiple regression that addresses the variation in a multivariate response matrix (in our case, composition based on incidence, abundance and biomass) and one or more matrices of explanatory variables (in our case, topographic and spatial variables) [55].

Spatial variables were obtained using the PCNM (Principal Coordinates of Neighbourhood Matrix) method [55]. The PCNM is based on Principal Coordinate Analysis obtained from a geographic distance matrix. The eigenvectors (axes) obtained from this analysis are called PCNMs, are uncorrelated and represent different spatial patterns, from coarse (axes with higher eigenvalues) to more refined (axes with smaller eigenvalues) [55,56]. In this paper, we extracted the spatial variables (PCNMs) from a Euclidean distance matrix between plots, which were represented by eigenvectors with positive eigenvalues and with spatial autocorrelation according to Moran’s I index [57]. For the analyses, we selected variables using the forward selection method to evaluate only the environmental and spatial variables that were more related to the studied metacommunity.

We assessed the following fractions: the environmental (topography) component independent of the space (a), environmental component inseparable of the spatial component (b), spatial component independent of the environment (c) and component not explained (d). As the coefficient of determination (R^2^) is influenced by the sample size and number of predictor variables, we used the adjusted R^2^ to determine the importance of each assessed fraction [58]. We performed the analyses in the computing environment R version 2.13 [59], associated with the PCNM package [60] for obtaining the spatial variables, Packfor [61] for variable selection and Vegan [62] for multiple regression and RDA.

## Results

**Fig 2:**
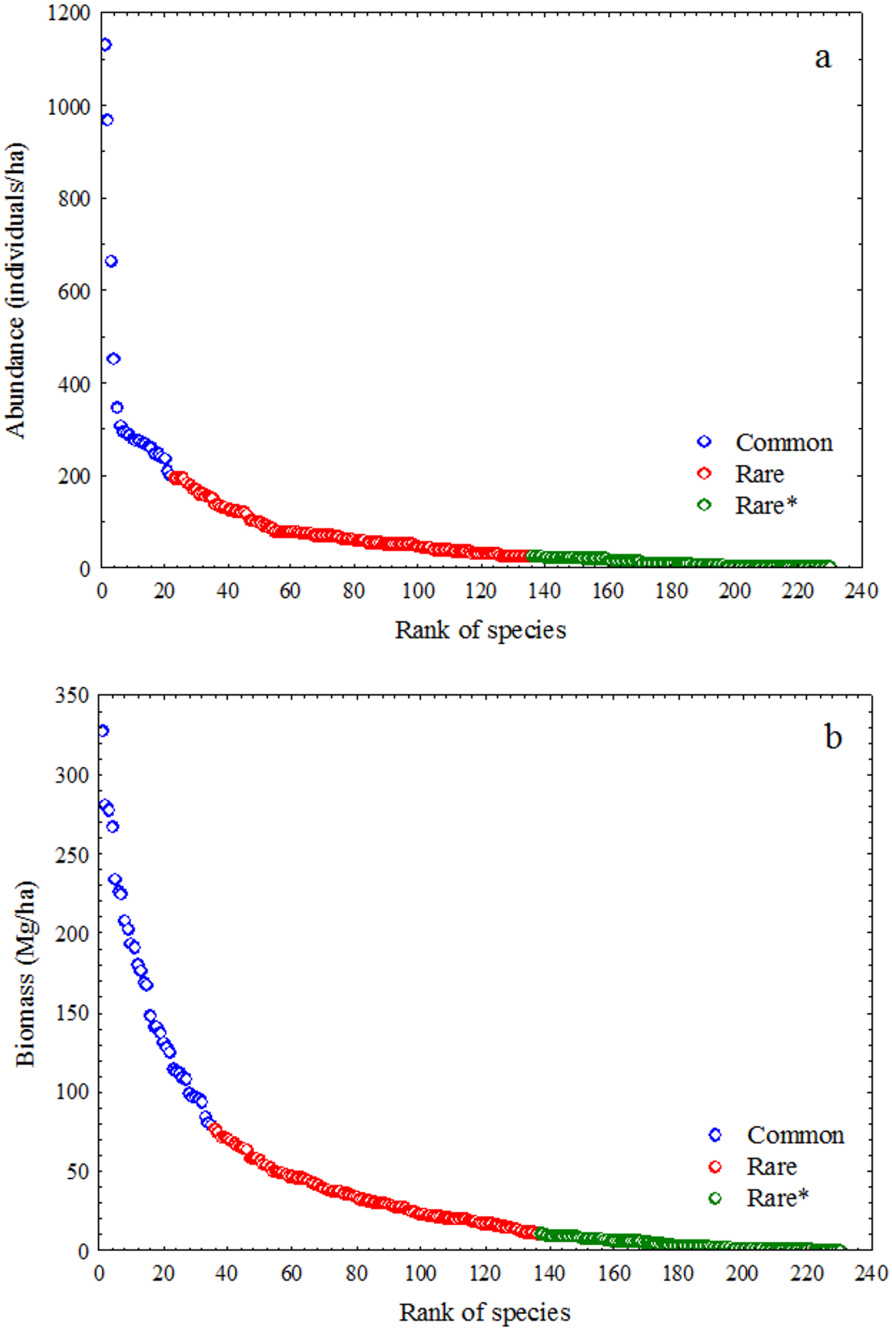
Rank of abundance (a) and rank of biomass (b) of Amazonian tree species of a metacommunity in the Tapajós National Forest, Pará State, Brazil. * indicates the rare species with the same information content as common species.

The results revealed that when considering the inflection points of the abundance curves, 22 species were considered common, and 208 species were considered rare (Fig 2a). The 93 rarest species had the same information content as the 22 most common species. When the inflection of the curves of species biomass was considered, 35 species were considered common, and 195 were considered rare (Fig 2b). In this case, the 94 rarest species had the same information content as the 35 most common species.

### Standardised richness, abundance and total biomass

Total standardised richness (without the abundance effect) and standardised richness of rare species could not be explained by any of the measured variables (topographic or spatial variables) (Table 2). Nevertheless, the standardised richness of common species was explained significantly by topography (Table 2). The total abundance was explained significantly by spatial variables (Table 2). Moreover, the abundance of common species was explained by both topographic and spatial variables, whereas rare species were explained just by the spatial variables (Table 2). The topography explains significantly the total tree biomass for all and for common species (Table 2). The total biomass of rare species could not be explained by any of the measured variables (Table 2).

**Table 2:** Results of the partial multiple regression and partial RDA with the coefficient of determination (R^2^) for whole community (total), common and rare species. Topography refers to the effects of geomorphometric variables without spatial component, shared refers to the effects of common variation between topographic and spatial components, and space refers to the spatial effects without topography. Common and rare species have the same information content and were delimited based on the inflection point of the species x abundance curve (in the case of abundance) or species x biomass curve (in the case of biomass).

	Topography (%)	Shared (%)	Space (%)	Not explained (%)
*Std*. *Richness*				
Total	-	-	-	100.00
Common (1–22)	10.4*	-	-	89.6
Rare (137–230)	-	-	-	100.00
*Abundance*				
Total	-	-	25.9**	74.1
Common (1–22)	11.4**	-	10.3**	78.3
Rare (137–230)	-	-	26.3**	73.7
*Biomass*				
Total	27.4***	15.1	-	57.5
Common (1–35)	14.2**	12.5	-	73.3
Rare (136–230)	-	-	-	100.00
*C*. *Incidence*				
Total	2.8***	1.8	6.6***	88.8
Common (1–22)	2.7**	2.7	5.4***	89.2
Rare (137–230)	-	0.9	0.3ns	98.8
*C*. *Abundance*				
Total	6.2***	0.4	10.4***	83.0
Common (1–22)	5.1**	2.7	10.6***	81.6
Rare (137–230)	0.1ns	0.8	0.4ns	98.7
*C*. *Biomass*				
Total	3.1**	3.2	1.6*	92.1
Common (1–35)	4.0**	4.2	1.9ns	89.9
Rare (136–230)	-	-	-	100.00

Numbers in parentheses refer to the rank position of the species. Univariate attributes: 1) std. richness (standardised richness, residuals of regression between abundance and richness); 2) abundance (sum of the abundance of the species per plot); and biomass (sum of biomass of the species per plot). Multivariate attributes (species x plots): 1) C. Incidence (composition based on incidence); C. Abundance (composition based on abundance); and C. Biomass (composition based on biomass).

*p < 0.05.

**p < 0.01.

***p < 0.001.

ns non-significant.

### Composition (incidence, abundance and biomass)

Our data reveal that topographic and spatial variables explained significant proportions of the variability when the analysis was based on an incidence matrix and on an abundance matrix, both for total and common species (Table 2). In these cases, the fraction explained by spatial variables was larger than the fraction explained by environmental variables (Table 2). Topographic and spatial variables did not explain the variation of rare species. When the analysis was based on biomass, the variability was explained significantly by topographic (larger fraction) and spatial variables (smaller fraction) for total species, but by only topography for common species and by no factor for rare species (Table 2).

## Discussion

Our results show that different attributes (richness, abundance, biomass and composition based on incidence, abundance and biomass) used to study this metacommunity respond differently to environmental and spatial processes. Common and rare species differ in terms of biological traits [63–65] and how they relate to environmental and spatial components [35,36,66]. Thus, our expectation was that the role of environmental and spatial variables differed between common and rare species. This expectation is confirmed by our study, suggesting that common and rare species are subject to different processes.

The communities are composed of few common species and many rare species. Due to the greater number of individuals, common species interact strongly with the various components of the system. Therefore, a common question is whether the common species are sufficient to describe the attributes (e.g., richness, abundance and composition) of the whole community [51,66,67]. If this is the case, studies on communities could focus on common species, which are more easily sampled. Several studies have shown that the conclusions found for all species are equivalent to those found using only common species [66–68]. Our study only partially confirms this expectation and it adds complexity to the picture by showing that this depends on the analysed attribute. For example, for standardised richness, total abundance (univariate) and composition based on biomass, the results for common species differ from those obtained for all species. For total biomass (univariate) and compositions based on incidence and abundance, there is a correspondence between the results obtained for the total community and common species. Our data suggest that for these last attributes, it is possible to draw appropriate conclusions for an entire community based on only common species.

The results to standardised richness are in disagreement with other studies which have shown that the richness patterns (total community) can be predicted by the richness of common species [12,69]. In our study, topography only explained the variability of the richness of common species, and neither topographic nor spatial variables explained the variability of the richness of the total community or of rare species. According to Lennon et al. [66], the richness of common species can be more easily explained by simple environmental gradients when compared to the richness of rare species. As the richness of rare species can be associated with rare environments [36,66], it is harder to predict it. Thus, the absence of the effect of the environment on the richness of rare species may be due to no inclusion of important but difficult to measure environmental variables, which must be associated with uncommon niches [36]. In this context, part of the variation of the standardised richness of common species can be predicted by the environmental gradient (in our case, topography), which does not occur with rare species.

Total abundance (univariate) and biomass (univariate) were explained by different processes when the total community, common species and rare species were considered. For total abundance, in all combinations (total community, common and rare species), spatial variables explained part of the variability. These results show that spatial processes determine a part of the total abundance variation, suggesting that the effects of mass are relevant when abundance is considered. For common species, in addition to spatial processes, the environmental variables were also important. In the case of the total biomass of the entire community and of the common species, only environmental variables were relevant. The topography influences other extremely important variables, such as soil texture and the availability of nutrients and water [11,70,71], and this may explain the results found for biomass. Our observations may have practical consequences. For example, we have sought ways to predict and monitor biomass at larger scales, and the relationship with topography can help since it influences other extremely important variables to the vegetation structure. Therefore, topography can be a surrogate of several variables that are difficult to measure in building predictive models that facilitate the monitoring of biomass and carbon stocks in tropical forests.

The data presented here show that the ecological processes underlying composition differ between common and rare species, in agreement with the results of Tsang and Bonebrake [68], who studied the composition of butterflies. On the other hand, this result disagrees with other data for different organisms (e.g., aquatic macroinvertebrates [31,67] and macrophytes [33]), which show that common and rare species are governed by the same processes, in these cases, by processes related to the niche. Specifically for vegetation, Wang et al. [72], who studied the effects of topography on the species composition of a subtropical forest, also verified that environmental variables are important determinants of the variation of the composition of common and rare species. However, this relation was much weaker for rare species. Our initial hypothesis was that the composition of rare species could be explained by the environmental and/or by spatial variables. Our results refuted this hypothesis since none of the components (environmental or spatial) explained the variation of the composition of rare species. This result may be a reflection of stochastic factors and of the non-inclusion of specific variables important for rare species.

Our initial expectations to the composition (based on incidence, abundance and biomass) were that the niche-related processes were the most relevant to explain data variability and that the studied metacommunity would follow the species sorting (SS) model [28]. The results showed that both environmental and spatial variables were relevant to explain variability for both total and common species (except for biomass of common species, which was explained by only environmental processes). These results suggest that on the scale studied, the data fit the species sorting (SS) + mass effect (ME) models. The SS and SS + ME models have been the most frequently adjusted models for empirical data. For example, Cottenie [28] studied 158 metacommunities and found that 44% of them fit the SS model and that 29% the SS + ME model. These patterns (SS or SS + ME) have been confirmed by most studies since Cottenie [28]. It is important to note in our study that in the case of composition based on incidence and abundance, the spatial component was more important than environmental component. In this context, several studies have shown that spatial variables explain a relevant part of the variability in both tropical and temperate forests [73,74]. However, the inclusion of other important environmental variables could increase the percentage explained by the environment and reduce the importance of spatial variables, whose effects may be a reflection of mass effects and dispersion difficulties, as well as responses to spatially structured but not measured environmental variables [25].

The result that the compositions based on incidence and abundance were explained more by spatial variables than by environmental variables is in agreement with a study carried out in the Bolivian Amazon Forest [75]. Myers et al. [75], who studied two forests in different latitudes, found that beta diversity in a tropical forest was explained predominantly by spatial variables, while beta diversity in a temperate forest was mostly explained by environmental variables. These authors suggest that in megadiverse systems with many rare species, such as tropical forests, intraspecific aggregation is more related to the limitation of the dispersion; while in temperate forests with fewer species, intraspecific aggregation is more related to environmental filters. Therefore, intraspecific aggregation, which generates beta diversity, is influenced by different processes in rich and poor metacommunities [75]. We also suggest other explanation, as forests in different latitudes may differ in complexity, a possible result is that the same set of environmental variables explain a smaller fraction of beta diversity in tropical forests (more complex system) than in temperate forests (less complex system). Therefore, a greater proportion of important unmeasured environmental variables in more complex forests could also increase the fraction explained by spatial variables in tropical forests. In this context, we suggest that in tropical forests, the spatial component tends to be larger than the environmental component, at least when compositions are based on incidence and abundance. In the case of composition based on biomass, the fraction explained by the environment is greater than the fraction explained by spatial variables.

In this study, we found that much of the variability was not explained by the environmental (represented by the topography) or by spatial variables. This is a relatively common result in studies of metacommunities [17,19]. Two main factors can help explain this result: 1) there are many environmental variables in tropical forests that affect the biota, and often only one portion of them (in our case, topographic variables) is measured; 2) tropical forests have a large number of biotic interactions, which despite having the potential to affect the community, are impossible to measure to capture their complexity. De Caceres at al. [73], who studied tropical, subtropical and temperate forests, found that the unexplained fraction was negatively correlated with latitude. The proportion of unexplained variability is probably due to stochastic and unmeasured variables. Baldeck et al. [18] showed that in addition to topography, the inclusion of variables such as nutrients can improve a model’s explanatory power. Although the topography is a good substitute for other variables that are difficult to measure, the inclusion of additional relevant variables should decrease the proportion of unexplained variability in the model.

This study revealed that for total biomass (univariate) and for compositions based on incidence and abundance, there was a correspondence between the results obtained for the total community and for common species. The possibility of monitoring the variability of the tropical forests based on only common species is highly relevant, especially given the current high deforestation rates. The variation of the measured community attributes of common species was explained by topographic and/or spatial variables. None of the components explained the measured community attributes (except for the total abundance) of rare species. These results indicate that predicting the attributes of rare species tree communities from environmental and spatial variables is a considerable challenge. In summary, our data show that in general, depending on the attribute, environmental and/or spatial variables are important to explain the variability in tree metacommunities. However, there are still doubts regarding whether the spatial component and large fractions of unexplained variability in forest metacommunities are due to insufficient data or are a feature of these systems [18,74]. In this study, the spatial component was important for several community attributes demonstrating the importance of a metacommunity approach when attempting to understand the main ecological processes underlying the diversity of tropical forest communities.

## Authorization for the field work

The study was carried out in the Tapajós National Forest (TNF) and dendrometric measurements (diameter at breast height and height) as well as botanical identification of the trees were done, just inside of this area. The authorization to carry out the field work at TNF was provided by the Instituto Chico Mendes de Conservação da Biodiversidade-ICMBio/MMA (SISBIO n. 20591–1). This study did not involve endangered or protected species and no biological samples were taken.

## Supporting information

S1 Table Selected topographic and spatial variables for partial multiple regression and partial redundancy according to forward selection. (DOCX)
