# Correction: Drivers of metacommunity structure diverge for common and rare Amazonian tree species

**DOI:** 10.1371/journal.pone.0203251

**Published:** 2018-08-31

**Authors:** The PLOS ONE Staff

The first and fifth authors’ names are listed incorrectly in the citation. The correct citation is: Bispo PC, Balzter H, Malhi Y, Slik JWF, Santos JR, Rennó CD, et al. (2017) Drivers of metacommunity structure diverge for common and rare Amazonian tree species. PLoS ONE 12(11): e0188300. https://doi.org/10.1371/journal.pone.0188300

In the Funding section, the author Fernando D. Espírito-Santo’s initials are listed incorrectly. The sentence should read: FDES was supported by Natural Environment Research Council (NERC) grants (BIO-RED NE/N012542/1 and AFIRE NE/P004512/1) and Newton Fund (The UK Academies/FAPESP Proc. N°: 2015/50392-8 Fellowship and Research Mobility).
