# Supplementary material for: Drivers of metacommunity structure diverge for common and rare Amazonian tree species
Source: PLoS One. 2017 Nov 20;12(11):e0188300. doi: 10.1371/journal.pone.0188300 (PMC5695845; doi:10.1371/journal.pone.0188300)
Supplement: S1 Table — (DOCX) [file pone.0188300.s001.docx]

**S1 Table.** Selected topographic and spatial variables from Partial Multiple Regression and Partial Redundancy according to forward selection. Univariate attributes: 1) std. richness (standardised richness, residuals of regression between abundance and richness); 2) abundance (sum of the abundance of the species per plot); and biomass (sum of biomass of the species per plot). Multivariate attributes (species x plots): 1) C. Incidence (composition based on incidence); C. Abundance (composition based on abundance); and C. Biomass (composition based on biomass). Topographic variables: Elevation (*h*); Slope (*G*); Profile curvature (*kv*); Plan curvature (*kh*); Height above the nearest drainage (*HAND*). Spatial variables: Principal Coordinates of Neighbour Matrices (PCNM 1, PCNM 2, PCNM 3, PCNM 4, and PCNM 5).

|  | Topographic variables | Spatial variables |
| --- | --- | --- |
| *Std. Richness* |  |  |
| Total | None selected | None selected |
| Common (1-22) | *h, HAND* | None selected |
| Rare (137-230) | None selected | None selected |
|  |  |  |
| *Abundance* |  |  |
| Total | None selected | PCNM 2, PCNM 3 |
| Common (1-22) | *kh* | PCNM 4 |
| Rare (137-230) | None selected | PCNM 2, PCNM 3 |
|  |  |  |
| *Biomass* |  |  |
| Total | *h, G* | PCNM 1, PCNM 2 |
| Common (1-35) | *h* | PCNM 1 |
| Rare (136-230) | None selected | None selected |
|  |  |  |
| *C.* *Incidence* |  |  |
| Total | *h, HAND, kv* | PCNM 1, PCNM 2, PCNM 3, PCNM 4, PCNM 5 |
| Common (1-22) | *h, G* | PCNM 1 |
| Rare (137-230) | *G* | PCNM 1 |
|  |  |  |
| *C.* *Abundance* |  |  |
| Total | *h, HAND, kv* | PCNM 1, PCNM 2, PCNM 3, PCNM 4, PCNM 5 |
| Common (1-22) | *G, HAND, kv* | PCNM 1, PCNM 2, PCNM3, PCNM 5 |
| Rare (137-230) | *G, HAND* | PCNM 1 |
|  |  |  |
| *C. Biomass* |  |  |
| Total | *h, G* | PCNM 1, PCNM 2 |
| Common (1-35) | *h, G* | PCNM 1, PCNM2 |
| Rare (136-230) | None selected | None selected |
